# Adherent-invasive *E. coli* – induced specific IgA limits pathobiont localization to the epithelial niche in the gut

**DOI:** 10.3389/fmicb.2023.1031997

**Published:** 2023-02-23

**Authors:** Rika Tanaka, Jin Imai, Hitoshi Tsugawa, Karl Bil Eap, Masaki Yazawa, Motoki Kaneko, Masashi Ohno, Kohei Sugihara, Sho Kitamoto, Hiroko Nagao-Kitamoto, Nicolas Barnich, Masashi Matsushima, Takayoshi Suzuki, Tatehiro Kagawa, Yasuhiro Nishizaki, Hidekazu Suzuki, Nobuhiko Kamada, Katsuto Hozumi

**Affiliations:** ^1^Department of Immunology, Tokai University School of Medicine, Isehara, Japan; ^2^Division of Gastroenterology, Department of Internal Medicine, Tokai University School of Medicine, Isehara, Japan; ^3^Department of Clinical Health Science, Tokai University School of Medicine, Isehara, Japan; ^4^Transkingdom Signaling Research Unit, Division of Host Defense, Tokai University School of Medicine, Isehara, Japan; ^5^Division of Gastroenterology, Shiga University of Medical Science, Otsu, Japan; ^6^Division of Gastroenterology, Department of Internal Medicine, University of Michigan, Ann Arbor, MI, United States; ^7^WPI Immunology Frontier Research Center, Osaka University, Suita, Japan; ^8^UMR1071 Inserm/University Clermont Auvergne, INRAE USC2018, M2iSH, CRNH Auvergne, Clermont-Ferrand, France

**Keywords:** inflammatory bowel disease, Crohn’s disease, host-pathogen interaction, IgA, adherent invasive E coli, pathobiont

## Abstract

**Background and aim:**

Adherent-invasive *E. coli* (AIEC) has been identified as a pathobiont associated with Crohn’s disease (CD), that prefers to grow in inflammatory conditions. Although the colonization by AIEC is implicated in the progression of the disease and exacerbates inflammation in murine colitis models, the recognition and response of host immunity to AIEC remains elusive.

**Methods:**

Antibiotic treated female C57BL/6 mice were inoculated by commensal *E. coli* and LF82 AIEC strains. Luminal-IgA fractions were prepared from feces and their binding to AIEC and other strains was assessed to confirm specificity. IgA binding to isogenic mutant strains was performed to identify the functional molecules that are recognized by AIEC specific IgA. The effect of IgA on epithelial invasion of LF82 strain was confirmed using *in vitro* invasion assay and *in vivo* colonization of the colonic epithelium.

**Results:**

Persistent colonization by AIEC LF82 induced secretion of luminal IgA, while commensal *E. coli* strain did not. Induced anti-LF82 IgA showed specific binding to other AIEC strains but not to the commensal, non-AIEC *E. coli* strains. Induced IgA showed decreased binding to LF82 strains with mutated adhesin and outer membrane proteins which are involved in AIEC – epithelial cell interaction. Consistently, LF82-specific IgA limited the adhesion and invasion of LF82 in cultured epithelial cells, which seems to be required for the elimination in the colonic epithelium in mice.

**Conclusion:**

These results demonstrate that host immunity selectively recognizes pathobiont *E. coli*, such as AIEC, and develop specific IgA. The induced IgA specific to pathobiont *E. coli*, in turn, contributes to preventing the pathobionts from accessing the epithelium.

## Highlights

– What is already known?

Persistent gut colonization by LF82, a human adherent-invasive *E. coli* strain, exacerbates intestinal chronic inflammation or fibrosis, however it is unknown how AIEC is recognized by host immunity.

– What is new here?

Persistent AIEC LF82 colonization induced the production of specific IgA that selectively recognized pathobionts and inhibited the adhesion and gut epithelial invasion of LF82 by targeting bacterial molecules involved in epithelial cell interaction.

– How can this study help patient care?

We believe that elucidating the host immune response against pathobionts will lead to the discovery of new targets for IBD therapy.

## Introduction

Inflammatory bowel disease (IBD) is a chronic inflammatory disorder of the gastrointestinal tract, which includes Crohn’s disease (CD) and ulcerative colitis (UC; Molodecky et al., 2012). Although the detailed etiology of IBD remains unclear, genetic and environmental factors, including the gut microbiota, diet, stress, and smoking cooperatively promote abnormal immune responses, leading to intestinal inflammation (Ananthakrishnan, 2015). Several reports have demonstrated that the gut microbiota plays a crucial role in the onset and progression of IBD. Patients with IBD display perturbed intestinal microbial communities, a condition referred to as gut dysbiosis (Sartor and Wu, 2017), which is defined by the decreased diversity of bacterial species. Moreover, it is considered that the balance between non-pathogenic commensals and potentially pathogenic microorganisms is disrupted in IBD-associated gut dysbiosis (Kamada et al., 2013; Ni et al., 2017).

The discovery of pathogenic bacteria led to understanding the etiology of infectious diseases, as stated in Koch’s principle. However, sequence-based studies on symbiotic bacteria have shown positive effects on human health, presenting a new bacteria-host relationship. Among such bacteria, a new category called pathobionts, that promotes disease only when host genes or environmental conditions are altered, has been identified. A pathotype of *Escherichia coli*, namely adherent-invasive *E. coli* (AIEC), has been identified as a potential pathobiont associated with IBD, particularly CD (Darfeuille-Michaud et al., 2004). AIEC exists in the ileal mucosa of CD patients and relies on its ability to replicate in the intestinal epithelial cells (Boudeau et al., 1999). Although AIEC colonization results in only mild inflammation in normal mice (Small et al., 2013), it exacerbates dextran sulfate sodium (DSS)- or enteric pathogen-induced colitis (Carvalho et al., 2008; Small et al., 2016). This is due to the shift in its metabolism to catabolize L-serine in the inflamed gut, in order to maximize its growth potential (Kitamoto et al., 2020). Furthermore, AIEC plays a crucial role in the development of intestinal fibrosis, one of the most severe complications of CD, *via* IL-33/ST2 signaling (Imai et al., 2019). Although the presence of AIEC plays an important role in the pathogenesis of CD, it is unknown how AIEC is recognized by host immunity. In this study, we demonstrated that persistent gut colonization in female C57BL/6 mice by an AIEC LF82 strain, but not by a commensal *E. coli* strain HS, induced luminal IgA against bacteria. The LF82 specific luminal IgA, which recognizes distinctive surface molecules associated with the pathogenicity of AIEC, limite7d the motility and invasion of the pathobiont into the epithelium, suggesting that IgA can discriminate AIEC from commensal *E. coli* and contributes to preventing the efficient colonization of the gut by pathobionts.

## Materials and methods

### Animals

Seven-to eight-week-old, specific pathogen-free (SPF) wild-type female C57BL/6 mice were purchased from CLEA Japan Inc. (Tokyo, Japan). Male mice may fight and injure themselves. To avoid the bacterial infection through the injured skin, female mice were used in this study. Mice were maintained in a specific pathogen-free (SPF) barrier facility at the Tokai University School of Medicine. All experiments were approved by the Institutional Animal Care and Use Committee at Tokai University (Kanagawa, Japan) (approval numbers: 191052, 202043, and 213037).

### Bacterial strains

*Escherichia coli* strains used in this study include the human commensal *E. coli* strain HS (Rasko et al., 2008), AIEC strains LF82, LF16, LF31, and LF73 (Darfeuille-Michaud et al., 2004), and non-AIEC-type LF1, LF6, and LF19 strains isolated from CD patients (Imai et al., 2019). Mutant LF82 strains used in the present study include Δ*fimH*, Δ*fliC*, Δ*ompA*, and Δ*ompC*. All bacterial strains were provided from Nicolas Barnich and Nobuhiko Kamada. All bacteria were grown at 37°C with shaking in Luria-Bertani (LB) broth.

### *E. coli* colonization in mice

SPF C57BL/6 mice were pre-treated with 20 mg of streptomycin (Strep) and ampicillin (Amp) at 1 g/L in drinking water for 1 week, 2 days prior to inoculation. *E. coli* strains LF82 or HS (1 × 10^9^ CFU/mouse) were inoculated into Strep/Amp-treated mice. To assess the bacterial counts in the colon, colorectal tissues samples were collected and homogenized in PBS with a Biomasher II (Nippi, 320–103). Serial dilutions of the homogenates were plated on the LB agar containing 50 μg/ml Amp and colony-forming units were counted.

### Immunohistochemistry

For immunohistochemistry analysis, colorectal tissue samples from mice infected with *E. coli* strains LF82 for 5 or 28 days were collected, fixed in 10% formaldehyde overnight, and embedded in paraffin. Tissue sections (3 μm) were depleted of paraffin, rehydrated in a series of graded ethanol solutions, and were subjected to antigen retrieval by heating for at 105°C in Target Retrieval Solution, pH 9 (Dako, S2375) for 10 min. The tissue sections were then incubated with antibody against *E. coli* (Abcam, ab137967) and Rhodamine Phalloidin (Invitrogen, R415) at 4°C overnight and then simultaneously with 4′,6-diamidino-2-phenylindole (DAPI; 1 μg/ml) and an Alexa Fluor 488-conjugated anti-rabbit secondary antibody for 1 h. Fluorescence images were captured with 200x magnification. Using an LSM700 confocal microscope (Carl Zeiss, Oberkochen, Germany). For the immunostaining of the cultures, *E. coli* strains LF82-infected Caco-2 cells were fixed in 4% paraformaldehyde overnight, and embedded in paraffin. The paraffin-processed cell line sections (3 μm) were depleted of paraffin, rehydrated in a series of graded ethanol solutions, and the paraffin-processed cell line sections were subjected to antigen retrieval by heating for at 105°C in Target Retrieval Solution, pH 9 (Dako, S2375) for 10 min. The paraffin-processed cell line sections were then incubated with antibody against *E. coli* (Abcam, ab137967) and Rhodamine Phalloidin (Invitrogen, R415) at 4°C overnight.

### Preparation of fecal IgA

Fecal pellets collected from each mouse were placed in PBS (100 mg/ml), homogenized, and then centrifuged (50 × *g*, 15 min, 4°C) to remove large particles. Supernatants containing fecal bacteria were further centrifuged (8,000 × *g*, 5 min, 4°C) to remove the bacteria, and the resulting supernatants were passed through a filter (0.22 μm) and collected as fecal IgA fraction.

### Flow cytometry

A single colony of LF82, its mutants, or HS *E. coli* was inoculated and cultured at 37°C for 18 h in a standard shaking incubator. Aliquots from the cultures containing 10^5^ CFU were centrifuged (8,000 × *g*, 5 min, 4°C) and suspended in PBS (0.1 ml). The fecal IgA fraction (50 ml) was added to the suspension and incubated for 30 min on ice, washed twice with PBS, and treated with PE-labeled anti-mouse IgA (mA-6E1, Thermo Fisher Scientific) for 30 min on ice. After washing, the bacterial pellet was fixed with PFA (4% in PBS) for 18 h, rewashed, suspended in PBS with DAPI (12.5 μ g/ml; Sigma-Aldrich), and analyzed using FACS Fortessa (BD Bioscience).

### Enzyme-linked immunosorbent assay

Total IgA in fecal samples were measured by Mouse IgA ELISA Quantitation Set (E90-103, Bethyl Laboratories) according to the manufacture’s instructions. Briefly, 96-well ELISA plates (Corning) were coated overnight at 4°C with anti-mouse IgA (A90-103A). Plates were washed and blocked with 3% BSA-PBS before diluted fecal samples were added, and incubated at 37°C for 1 h. After washing, mouse IgA was detected using HRP-conjugated anti-mouse IgA (A90-103P), and OPD peroxidase substrate. After 15–20 min, the reaction was quenched with 3 N H_2_SO_4_ stop solution. Purified mouse IgA (14-4762-81, Invitrogen) served as standard. Absorbances at 492 nm were measured on a tunable microplate reader (VersaMax, Molecular Devices).

### *E. coli* infection of Caco-2 cells

Caco-2 cells (ECACC, 86010202) were grown into 6-or 24-transwell insert plates (Thermo, 140,660 and Griner bio-one, 662,641) at 1 × 10^6^ or 5 × 10^5^ cells per well for 7–10 days, in DMEM. *E. coli* strains LF82 were pre-cultured in DMEM at 37°C for 3 h, and then the bacteria (1 × 10^2^ bacteria) were incubated with fecal IgA (30 μg protein) at 4°C overnight. Caco-2 cells were incubated with fecal IgA-treated *E. coli* strains LF82 (1 × 10^2^ bacteria) for 3 h, and then washed three times with PBS and incubated in DMEM containing 100 μg/ml gentamycin for 3 h to kill the extracellular bacteria. The cells were washed with PBS and lysed with PBS containing 1% Triton X-100. The cell lysates were plated on LB agar, and surviving intracellular *E. coli* strains LF82 were enumerated as colony-forming units.

### Statistical analyses

Statistical analyses were performed using GraphPad Prism software (version 5.0; GraphPad Software, Inc., San Diego, CA). Differences between two groups were evaluated using 2-way analysis of variance (parametric), followed by the Bonferroni correction for parametric samples, or the Dunn’s test for nonparametric samples as a *post hoc* test. Statistical significance was set at *p* < 0.05.

All authors had access to the study data and had reviewed and approved the final manuscript.

## Results

### AIEC colonization persists after antibiotics are administrated.

**Figure 1:**
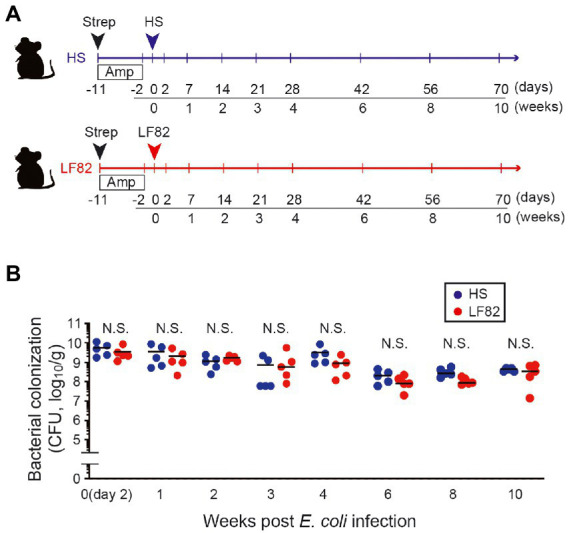
AIEC colonization persists after antibiotics are administrated. **(A)** SPF female C57BL/6 mice were treated with 20 mg streptomycin (Strep) followed by 1 g/L ampicillin (Amp) in drinking water for 9 days. Two days later, Strep/Amp-treated mice were inoculated with human commensal *E. coli* strain HS or human AIEC strain LF82 (1 × 10^9^ CFU/mouse). **(B)** Intestinal colonization (in feces, log_10_/g) of *E. coli*. Dots represent individual mice. Bars indicate median. N.S., not significant by 2-Way ANOVA with Bonferroni post-hoc test.

To examine how host immunity recognizes colonization by AIEC in mice, a murine model in which these bacteria are maintained for a long duration was established. As previously reported (Imai et al., 2019; Oberc et al., 2019), treatment with streptomycin strongly enhanced early AIEC infection. However, the inoculated bacteria were expelled within a week and did not persist in the mice. Another round of antibiotic treatment where ampicillin was added in drinking water (Kim et al., 2018; Miyauchi et al., 2020), was effective for their persistence. Following antibiotic treatment, mice were orally inoculated with either a CD patient-derived AIEC strain LF82(Darfeuille-Michaud et al., 2004) or the human commensal *E. coli* strain HS (Figure 1A). No *E. coli* growth was observed in antibiotic-treated control mice (without human *E. coli* inoculation; Supplementary Figure 1, < 2×10^2^ CFU/g feces), suggesting that antibiotic treatment did not induce the blooms of resident *E. coli* in mice. Therefore, we believe that the *E. coli* grown in LF82-or HS-colonized mice are likely to be inoculated exogenous *E. coli* strains. However, we did not verify whether these growing *E. coli* colonies are identical to the inoculated strains. In contrast, > 10^9^ CFU/g feces were observed in LF82-or HS-inoculated mice (Figure 1B). Colonization by the inoculated human *E. coli* strains persisted at 10^7^ to 10^9^ CFU/g feces even 10 weeks after inoculation (Figure 1B). Thus, we used this protocol and examined the host response against persistent infection by human-derived *E. coli* strains.

### Persistent colonization by AIEC induced AIEC-specific IgA secretion

**Figure 2:**
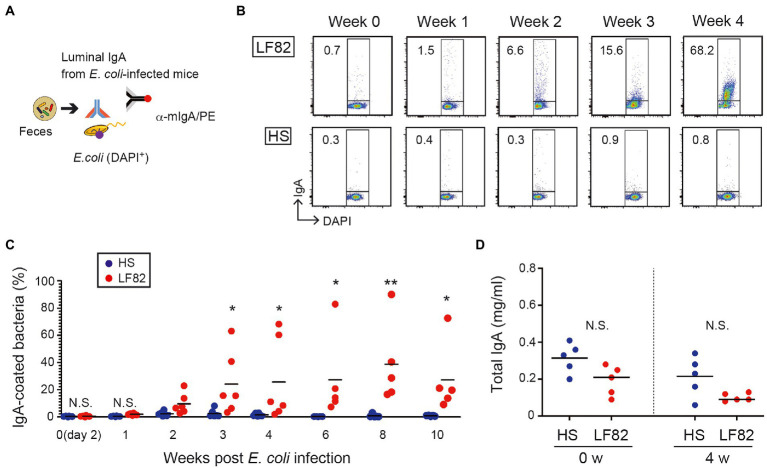
Persistent colonization of AIEC induces bacterial specific IgA but not non-AIEC colonization. **(A)** Luminal IgA (L-IgA) was prepared from feces of infected mice every week and analyzed for their ability to bind to bacteria. **(B)** The production of L-IgA in response to LF82 (upper panels) or HS (lower panels) was analyzed by flow cytometry. The numbers in the panels indicate the percentage of IgA^+^ fraction within the DAPI^+^ total bacteria. **(C)** The kinetics of IgA production in infected mice. The binding of each IgA fraction to bacteria was determined as shown in a. **(D)** Total IgA concentration was assessed by ELISA (*N* = 5). IgA fractions were prepared from feces of before (0 week) and after infecting the mice (4 weeks). Bars represent median. N.S., not significant; **, *p* < 0.01; *, *p* < 0.05 by Mann–Whitney U test and one-Way ANOVA with Bonferroni post-hoc test.

The consequence of the persistent colonization by AIEC was assessed with respect to the reaction of the host’s immune system to the pathogen. As previously reported (Palm et al., 2014), colitogenic members of the intestinal microbiota were highly coated with IgA in mice. We examined whether host immunity responds to AIEC through the production of specific IgA. Luminal IgA (L-IgA) were prepared from the feces of infected mice every week and analyzed for their ability to bind to bacteria (Figure 2A). IgA that recognizes AIEC LF82 was absent during early infection (1 week) and was induced 2 weeks after infection (Figures 2B,C). Even after 10 weeks post infection, significant population of LF82 was coated by fecal IgA (Figures 2B,C). We have never seen any pathogenic phenotype only with the infection of LF82 AIEC even in the mice with the elevated anti-LF82 IgA. In contrast, commensal *E. coli* strain HS colonization did not induce HS-specific IgA response despite its high level of colonization (Figures 2B,C). This strain lives away from the epithelial layer (Sevrin et al., 2020), resulting in the ignorance of the specific immune system. The concentration of total IgA was not different in early (2 days) and late (4 weeks) colonization by *E. coli* strains (Figure 2D). These results suggested that the host recognizes the pathobiont and produces specific IgA upon persistent colonization.

### AIEC-specific IgA can distinguish AIEC from human commensal bacteria

**Figure 3:**
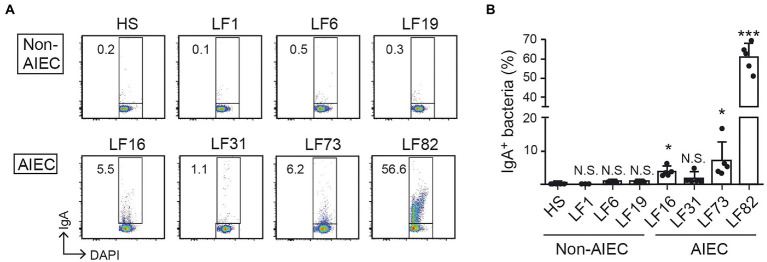
AIEC-specific IgA distinguishes AIEC from human commensal bacteria. The IgA of LF82-infected mice 4 w after inoculation was analyzed for its binding ability to several AIEC or non-AIEC strains by flow cytometry. **(A)** Representative flow cytometry plots and **(B)** percentages of IgA-bound bacteria. Left bars indicate non-AIEC strains and right bars indicate AIEC strains. Data shown are mean ± SD (*N* = 5). N.S., not significant; ***, *p* < 0.001; *, p < 0.05 by Dunnett’s test (vs. HS).

To further examine the extent to which anti-AIEC IgA binds pathobiont *E. coli* selectively, we evaluated IgA reactivity. Luminal IgA was harvested from mice colonized by LF82 (28 days) and used for the detection of various AIEC and non-AIEC strains. IgA isolated from LF82-colonized mice bound human-derived AIEC strains (Figures 3A,C) but did not bind commensal non-AIEC human-derived *E. coli* strains (Figures 3A,B). These results indicated that the luminal IgA induced by the LF82 colonization does not recognize the *E. coli* common antigens that are shared by AIEC and non-AIEC strains (e.g., bacterial cell wall components such as lipopolysaccharide or flagellin). Rather, the induced IgA recognizes AIEC-specific antigens that are likely associated with the pathogenicity of AIEC.

### LF82-induced IgA recognizes the functional molecules in AIEC, which contribute to the interaction with the intestinal epithelium.

**Figure 4:**
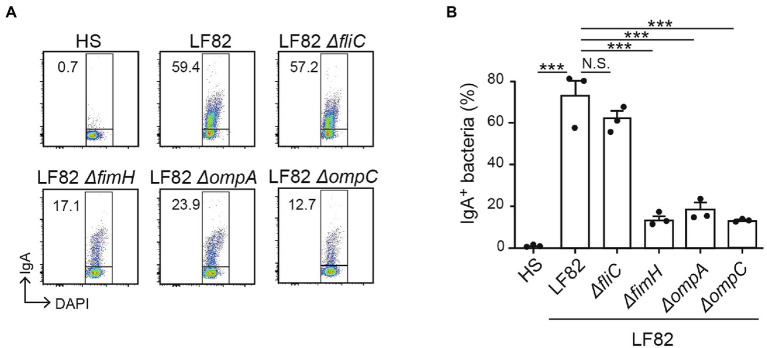
LF82-specific IgA recognizes molecules associated with its pathogenicity. The reactivity of IgA from LF82-infected mice to several LF82-derived mutants (*∆fliC*, *∆fimH*, *∆ompA*, and *∆ompC*) was analyzed by flow cytometry. **(A)** Representative flow cytometry plots and **(B)** percentages of IgA-bound bacteria. Data shown are mean ± SD (*N* = 3). N.S., not significant; ***, *p* < 0.001 by Dunnett’s test (vs. LF82).

We sought the bacterial molecules that are recognized by the luminal IgA induced by the persistent colonization of AIEC. We employed isogenic mutant strains of LF82 lacking FimH (adhesin; Dreux et al., 2013), FliC (flagellar component; (Barnich et al., 2003). OmpA, or OmpC (outer membrane proteins; Rolhion et al., 2007). Consistent with the previous experiment, luminal IgA induced by the colonization of AIEC LF82 bound WT LF82 but not commensal *E. coli* HS (Figure 4). Compared to WT LF82, the LF82 *fliC* mutant showed the same levels of IgA binding (Figure 4). This result indicated that flagella protein, encoded by the *fliC* gene was not recognized by the luminal IgA induced by the AIEC colonization. In contrast, *fimH*, *ompA*, and *ompC* mutant LF82 strains showed a low rate of IgA binding compared to WT LF82 (Figure 4). FimH and OmpA/C are linked to the interaction and invasion of intestinal epithelium by AIEC. Thus, luminal IgA induced by the persistent colonization of AIEC recognizes the functional molecules associated with its pathogenicity.

### AIEC-specific IgA fraction inhibits the invasion of AIEC into epithelial layers *in vitro*

**Figure 5:**
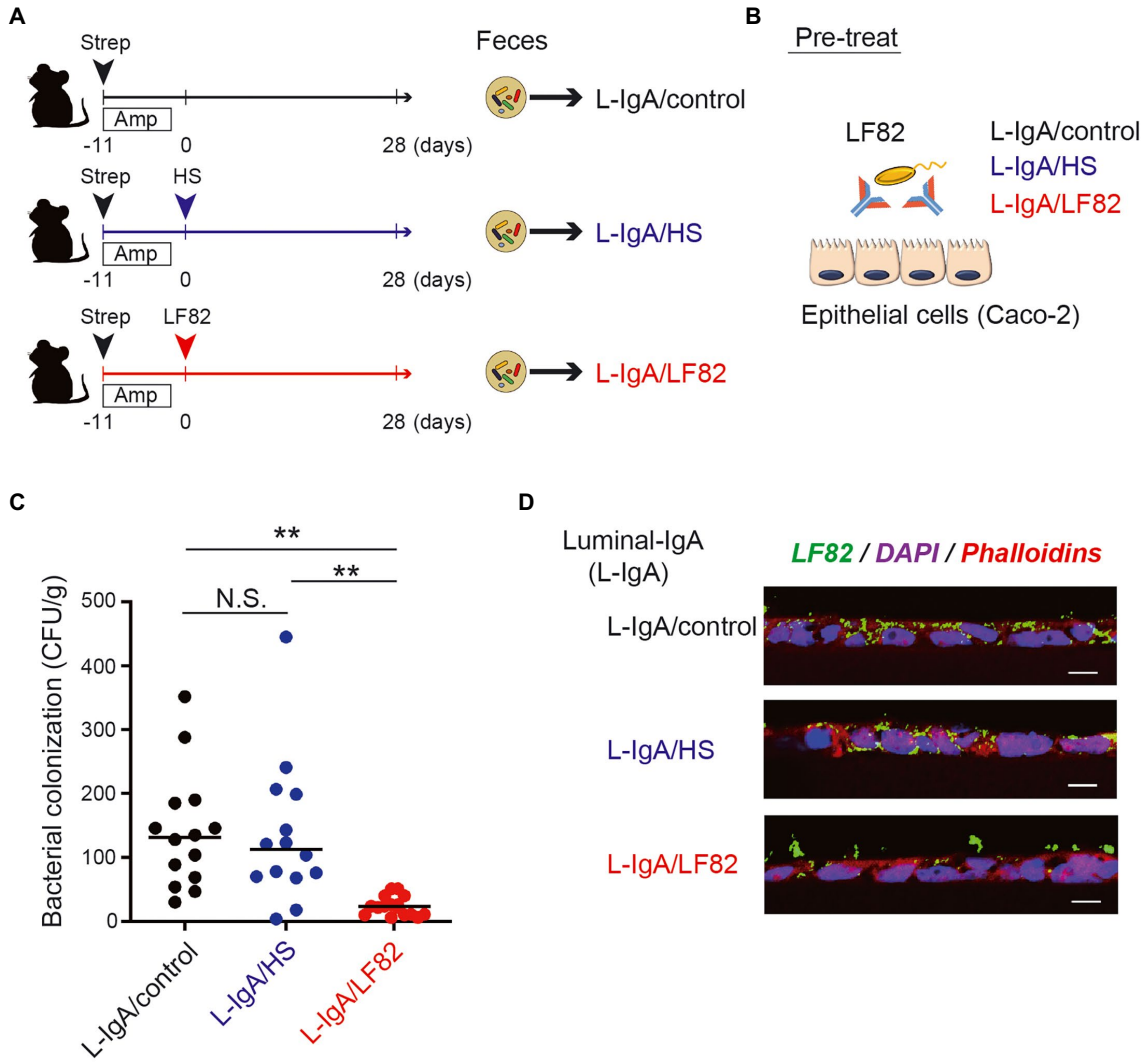
AIEC-specific IgA fraction inhibits the invasion of AIEC into the epithelial layers *in vitro*. **(A)** The IgA fractions containing luminal IgA (L-IgA) were obtained from the mice infected with HS (L-IgA/HS), LF82 (L-IgA/LF82) or without infection (L-IgA/Control), 28 days after inoculation. **(B)** For the invasion assay, LF82 was pre-treated with each of the IgA fractions, and its ability to invade the layer of human epithelial line, Caco-2 was examined. **(C)** The invasion ability of LF82 pretreated with L-IgA was assessed by the number of bacteria that resided in the epithelial layers. **(D)** Immunofluorescence images of the epithelial layers incubated with LF82 which was pre-treated with L-IgA from control (L-IgA/Control), HS-infected (L-IgA/HS) or LF82-infected (L-IgA/LF82) mice are shown. LF82 was visualized by the staining with anti-*E.coli* Ab (LF82, green), and the nucleus and cytoplasm of Caco-2 cells were, respectively, detected by the staining with DAPI (blue) and Phalloidin (red). Data shown are mean ± SD (*N* = 14). N.S., not significant; **, *p* < 0.01 one-Way ANOVA with Bonferroni post-hoc test.

To examine whether AIEC-specific IgA suppresses of the epithelial invasion of AIEC, we performed an *in vitro* invasion assay with the human epithelial cell line Caco-2. The IgA fractions containing luminal IgA (L-IgA) were obtained from the mice infected with HS (L-IgA/HS), LF82 (L-IgA/LF82) strains, 28 days after inoculation or the mice without infection (L-IgA/Control; Figure 5A). Prior to cocultivation with Caco-2, LF82 was pre-treated with each IgA fraction for 3 h, and its ability to invade the Caco-2 monolayer was examined (Figure 5B). LF82 showed frequent invasion into the mucosal epithelial layers compared to HS (Supplementary Figure 2). Interestingly, pre-treatment with L-IgA/LF82 compromised the invasion of LF82 into epithelial cells, while the pre-treatment with L-IgA/HS had no effect (Figure 5C). Moreover, using immunofluorescence, we evaluated the differences in the invasion of epithelial layers by LF82 pre-treated with each L-IgA. Only pre-treatment with L-IgA/LF82 impaired LF82 invasion into epithelial cells (Figure 5D) and might have led to their aggregation on the epithelial surface. These results demonstrated that AIEC-specific IgA prevents AIEC from attaching to and invading intestinal mucosal epithelial cells.

### The number of LF82 which invade the mucosal epithelium decreases at the late stage of the infection

**Figure 6:**
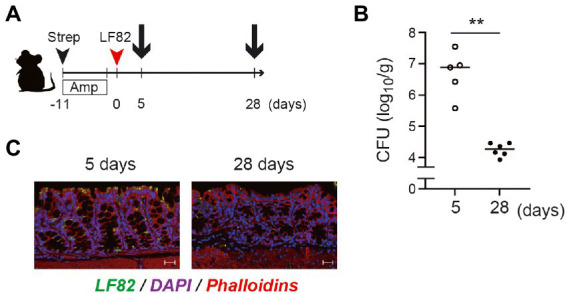
The number of LF82 bacteria which invade the mucosal epithelium decreases at the late stage of the infection. **(A)** The timing for the preparation of gut derived from LF82-infected mice is shown. **(B)** The invasion of LF82 into the mucosal epithelium at the early (5 days) and the late (28 days) stages was assessed by the number of bacteria in the gut. **(C)** Two representative immunofluorescence images are shown; left panel is the mucosal epithelium at 5 days and right panel is at 28 days. Data shown are mean ± SD (*N* = 5). **, *p* < 0.01 by Mann–Whitney U test.

To estimate the functional role of LF82-specific luminal IgA *in vivo*, we examined the number of mucosa-associated LF82 in the colon at the early and late stages of colonization (Figure 6A). On day 5 post colonization (before development of LF82-specific IgA), ~ 10^7^ CFU/g of LF82 were observed in the colonic mucosa (Figures 6B,C). In contrast, significantly lower number of LF82 were observed in the mucosal tissue on day 28 post colonization (i.e., after the induction of LF82-specific IgA; Figures 6B,C). Since there was no difference in the number of AIEC detected in the feces (Figure 1B), it is possible to realize that LF82-specific IgA binds to pathobiont, limiting its adhesion/invasion into the colonic epithelium.

## Discussion

We demonstrated that persistent colonization by a human pathobiont AIEC LF82 resulted in the induction and secretion of pathobiont-specific IgA in the intestine. The induced IgA was fairly specific to the pathobiont and did not recognize the common antigens shared with the commensal *E. coli*. It specifically reacted with pathogenic molecules harbored by AIEC strains. The IgA induced against LF82 prevented its adhesion and invasion into the epithelium. These findings suggested that adaptive immunity in mucosal tissues distinguishes the pathobionts from commensals and avoids responding to innocuous antigens, which is significant for the reduction of the pathogenicity.

Recent studies on the host-microbe relationship have revealed the existence of pathobionts involved in pathogenicity only under certain conditions, and their relevance to the development of chronic inflammatory diseases is attracting attention (Mazmanian et al., 2008). However, in some cases, only the sequence-based association of characteristic symbionts with diseases has been shown, and their relationship to the etiology of the diseases remains elusive (Jochum and Stecher, 2020). In this context, host responses, including specific IgA production to typical pathobionts, have been investigated. The first bacterium identified as a pathobiont was *Helicobacter hepaticus*, in murine microbiota, which induced intestinal inflammation only in immunocompromised mice (Cahill et al., 1997). *H. hepaticus*-specific T-cells differentiate into RORγt^+^ induced regulatory T (iTreg) cells, restraining pro-inflammatory Th17 cells in healthy conditions, but become colitogenic Th17 cells in disease-susceptible IL10-deficient conditions (Xu et al., 2018). Of note, *H. hepaticus* is also recognized by humoral immunity, producing specific IgA, in immunocompromised conditions (Friedrich et al., 2021). Consistent with this characterization, several additional pathobionts have been identified in the murine gastrointestinal tract. Segmented filamentous bacteria (SFB) are known to stimulate CD4+ Th17 differentiation(Ivanov, 2017) and can trigger intestinal inflammation along with a complex microbiota in severely immune-deficient mice (Stepankova et al., 2007). *Mucispirillum schaedleri* also induced colitis in a *Nod2/Cybb* deficient state, and is known to be associated with susceptibility to CD (Caruso et al., 2019). Interestingly, these pathobionts share unique characteristics, inhabit an unusual niche in close proximity to the intestinal epithelium (Fox et al., 1994; Palm et al., 2014), and induce T-cell-dependent specific IgA responses in mice (Ge et al., 2005; Palm et al., 2014; Friedrich et al., 2021). However, all these pathobionts were isolated from mice, and there is little validation of pathobionts isolated from human patients. Recently, it was reported that specific IgA response in mice infected with a human pathobiont *Helicobacter pylori*, is critical for protecting the stomach and eliminating the bacteria (Satoh-Takayama et al., 2020). In case of AIEC, the TLR4 and TLR5 mediated inflammatory responses due to recognition of these bacteria by the innate immune system have been reported (Mohammadi et al., 2019; Satoh-Takayama et al., 2020). However, the response of the adaptive immune system remains largely unknown. This study first uncovered the specific IgA response to AIEC, but not the related to commensal *E. coli* in mice. Since AIECs exhibit high adhesion to the intestinal epithelium similar to other pathobionts, our results suggest that IgA induction could be a common property of pathobionts across species.

AIECs have the ability to adhere to and invade intestinal epithelial cells. In this context, AIEC uses FimH located at the tip of type 1 pili, which interacts with the glycoprotein CEACAM6 on intestinal epithelial cells (Boudeau et al., 2001), for adhesion. This interaction mediates not only adhesion, but also invasion (Dreux et al., 2013). Thus, FimH could be the basis for the unique characterization of AIECs. In addition, OmpA and OmpC, the major proteins on the surface of outer membrane vesicles (OMVs), are involved in the invasive ability of AIEC by delivering several molecules to the epithelium (Rolhion et al., 2010). Notably, a specific IgA recognizing OmpC was frequently detected in the serum of CD patients (Mow et al., 2004), suggesting that OmpC possesses unique epitopes that are different from those in commensal bacteria. In contrast, though the expression of FliC protein, the major component of flagellum encoded by *the fliC* gene, was differently regulated, it is common between AIEC and commensal strains (Chervy et al., 2020). This indicated that the FliC protein was not specific to AIEC. Consistently, we showed here that IgA prepared from LF82-inoculated mice specifically reacted with wild-type LF82 and its *fliC* mutant, but showed attenuated reactivity to the *fimH* and *ompA/C* mutants. Thus, it could be confirmed that anti-LF82 IgA recognized the epitopes specific for AIEC. As IgA recognizes FimH or OmpA/C, which are strongly associated with AIEC adhesion and invasion, it may contribute to the specific removal of AIEC from the body through the gastrointestinal tract.

It is understood that secretory IgA (SIgA) can bind to microbes and restrict their motility which is necessary for their adhesion and invasion into the epithelium (immune exclusion), resulting in the prevention of infections (Huus et al., 2021). Maternal SIgA in breast milk is important to protect against microbial infections encountered during the neonatal period (Zheng et al., 2020). Thereafter, the developing infants continue to increase their own secretion of IgA to prevent the infection and maintain homeostasis of the commensal microbes (Planer et al., 2016). Of note, IgA-deficient patients exhibit a dysbiosis of the gut microbiota with reduced diversity and over-representation of pathobionts, suggesting that the luminal IgA is linked with the immune exclusion of pathobionts and regulates the homeostasis in the gut microbiota (Jørgensen et al., 2019). In this study, we showed that the number of invaded LF82 in the monolayer cultures of human epithelial cells *in vitro* decreased with the LF82-specific IgA. This seemed to be consistent with the reduction of LF82 on the colonic epithelium at the late stage of the infection, in which the LF82-specific luminal IgA was fully produced. These results suggest that the LF82-specific IgA prevents the pathobionts from accessing the epithelium and keeps them in the lumen, which should contribute to reducing their pathogenicity. However, our study has limitations in providing insight into the functional importance of pathobiont-specific IgA in the physiological and pathological situations. In our experimental system, LF82 was still detected in the feces at the late stage with the specific IgA, suggesting that detachment from the epithelium does not lead to the clearance from the body. The competitive environment with the usual commensals might be required for the complete exclusion. Moreover, it has never been examined how the specific IgA functions in chronic inflammation. Thus, the role of AIEC-specific IgA during the onset and progression of IBD should be validated in an AIEC-dependent chronic colitis model. We believe that elucidating the host immune response against pathobionts will lead to a better understanding of the etiology of IBD.

## Author’s note

Persistent gut colonization by LF82, a human adherent-invasive *E. coli* strain, induced the production of specific IgA that selectively recognized pathobionts and inhibited the adhesion and gut epithelial invasion of LF82 by targeting bacterial molecules involved in epithelial cell interaction.

## Data availability statement

The raw data supporting the conclusions of this article will be made available by the authors, without undue reservation.

## Ethics statement

The animal study was reviewed and approved by the Animal Experimentation Committee of Tokai University.

## Author contributions

RT, JI, HT, KE, and KH performed the experiments and RT, JI, HT, and KH wrote the manuscript. JI and KH organized the database and performed the statistical analysis. MY, MO, SK, NK, NB, MM, TS, YN, HS, and NK contributed to the conception and design of the study. MK, KS, TK contributed to the experiments for revise data. All authors contributed to the article and approved the submitted version.

## Funding

This work was supported by Japan Society for the Promotion of Science (19K17413, 22K042 to JI; 20K07330 to KH), Tokai University School of Medicine Research Aid (2019, 2020 to RT, 2022 to JI), Tokai University Supporters Association Research and Study Grant 2021 (JI), National Institutes of Health grants (DK108901 to NK), Crohn’s and Colitis Foundation (RFA 369259 and CDA 632826 to HN-K), The Department of Defense (CA191087 to SK) and the University of Michigan Center for Gastrointestinal Research Pilot Feasibility Project P30 (DK034933 to SK).

## Conflict of interest

The authors declare that the research was conducted in the absence of any commercial or financial relationships that could be construed as a potential conflict of interest.

## Publisher’s note

All claims expressed in this article are solely those of the authors and do not necessarily represent those of their affiliated organizations, or those of the publisher, the editors and the reviewers. Any product that may be evaluated in this article, or claim that may be made by its manufacturer, is not guaranteed or endorsed by the publisher.

## Abbreviations

AIEC: Adherent-invasive *E. coli*
CD: Crohn’s disease
IBD: Inflammatory bowel disease
UC: Ulcerative colitis
SPF: specific pathogen-free
