# Supplementary material for: Adherent-invasive E. coli – induced specific IgA limits pathobiont localization to the epithelial niche in the gut
Source: Front Microbiol. 2023 Feb 23;14:1031997. doi: 10.3389/fmicb.2023.1031997 (PMC9995611; doi:10.3389/fmicb.2023.1031997)
Supplement: Supplementary file 1 [file Data_Sheet_1.PDF]

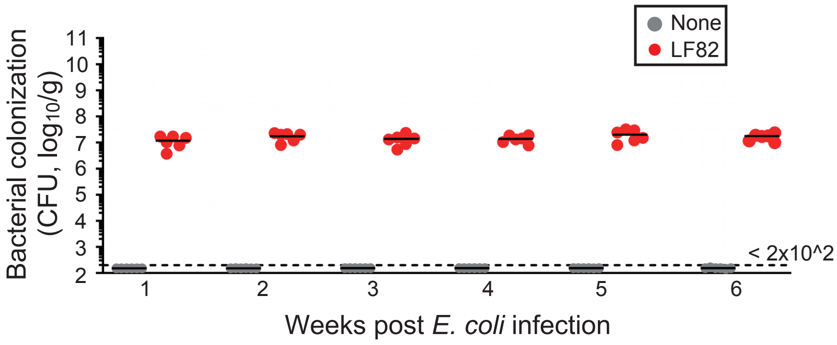

Figure S1. Few colonization was found without the innoculation of *E. coli*.

Antibiotics-treated mice were inoculated with human AIEC strain LF82 (LF82, n=6) or PBS (None, n=6) as shown in Figure 1. Dots represented Intestinal colonization (in feces) in indivisual mice.The detection limit in this assay is 2x10^2 CFU/g bacteria. Bars indicate median.
