# Supplementary material for: Adherent-invasive E. coli – induced specific IgA limits pathobiont localization to the epithelial niche in the gut
Source: Front Microbiol. 2023 Feb 23;14:1031997. doi: 10.3389/fmicb.2023.1031997 (PMC9995611; doi:10.3389/fmicb.2023.1031997)
Supplement: Supplementary file 2 [file Data_Sheet_2.PDF]

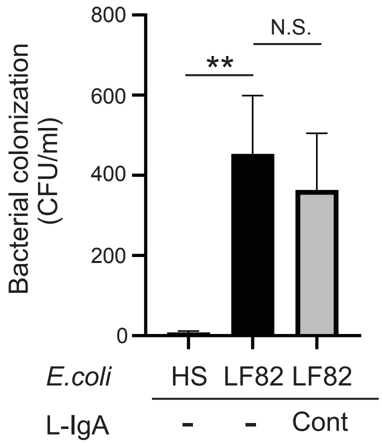

Figure S2. Characterization of AIEC and non-AIEC by the invasion assay in vitro.

The invasion ability of AIEC (LF82) and non-AIEC (HS) was assessed by an invasion assay as shown in Figure 5. AIEC (LF82) was able to invade the epithelial layers of Caco-2, human epithelial cells with or without (-) the luminal IgA fraction (L-IgA) prepared from control mice (Cont), in contrast to non-AIEC (HS) which did not adhere to or invade the epithelium in vitro. The number of bacteria remaining in the epithelium after antibiotic treatment was calculated. Data shown are mean ± SD (N=6).

N.S., not significant; **, $P < 0.01$ one-Way ANOVA with Bonferroni post-hoc test.
